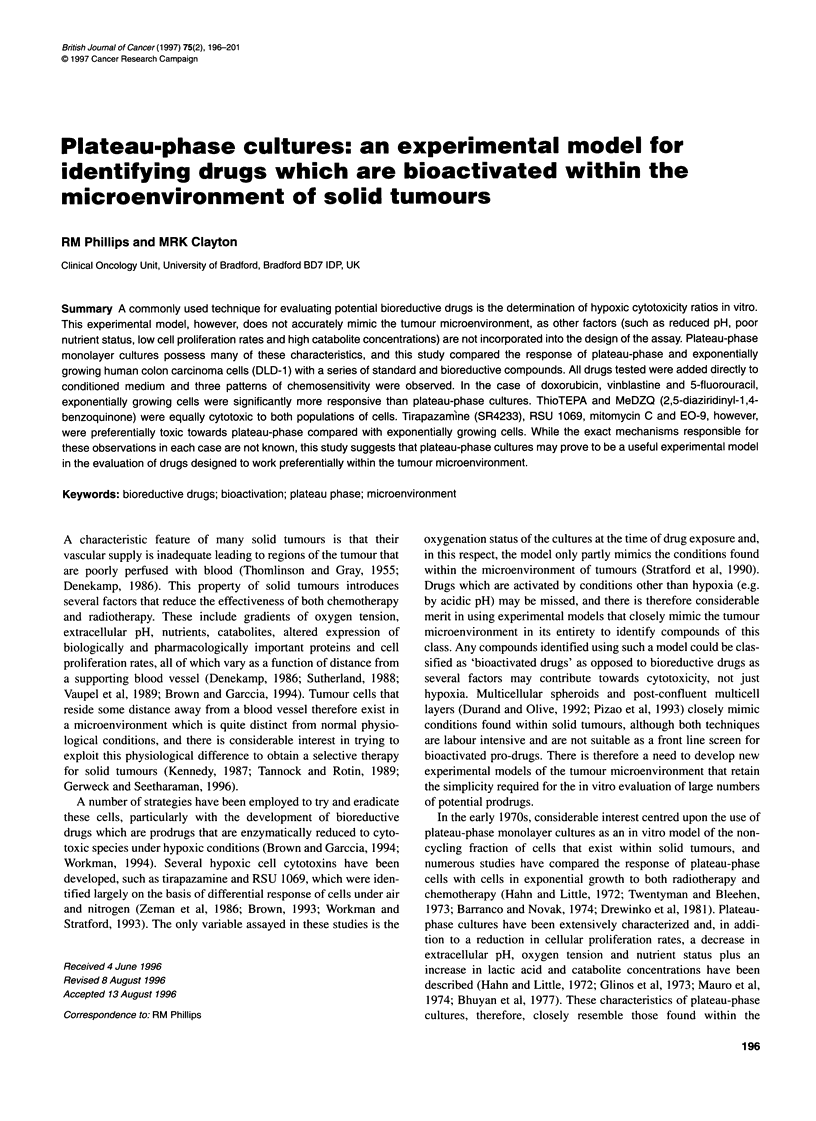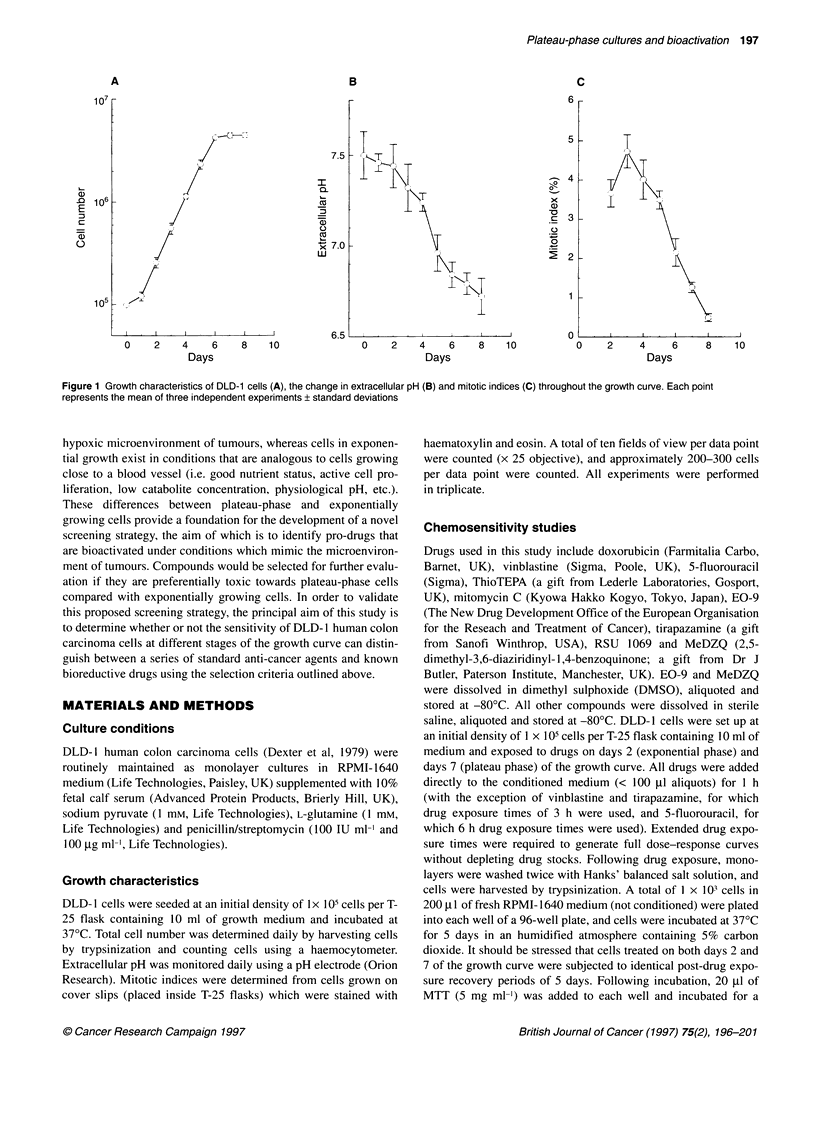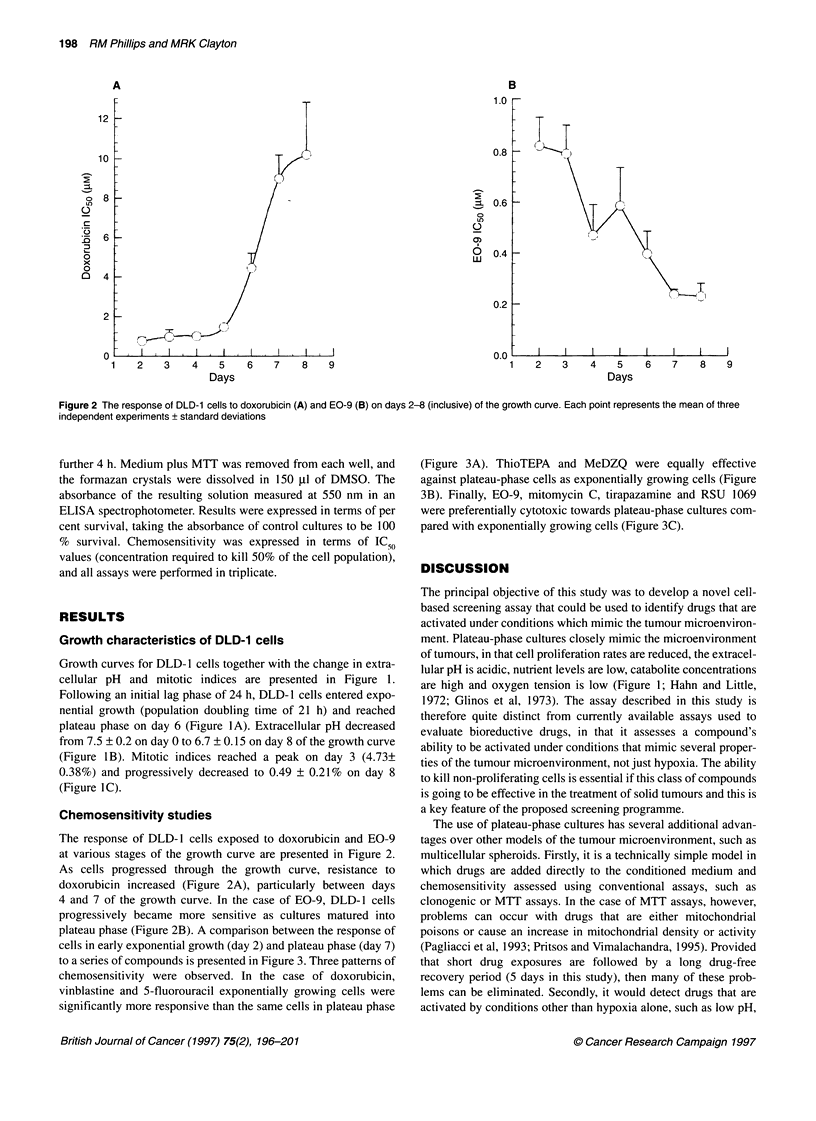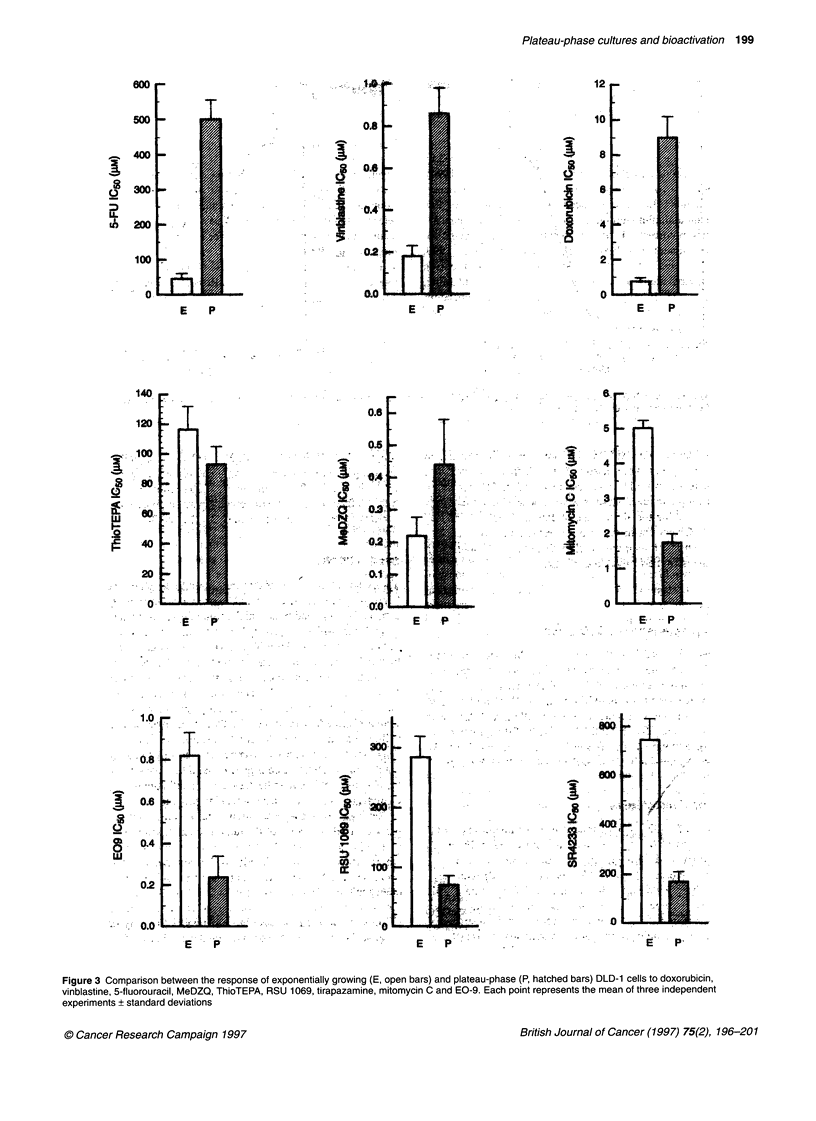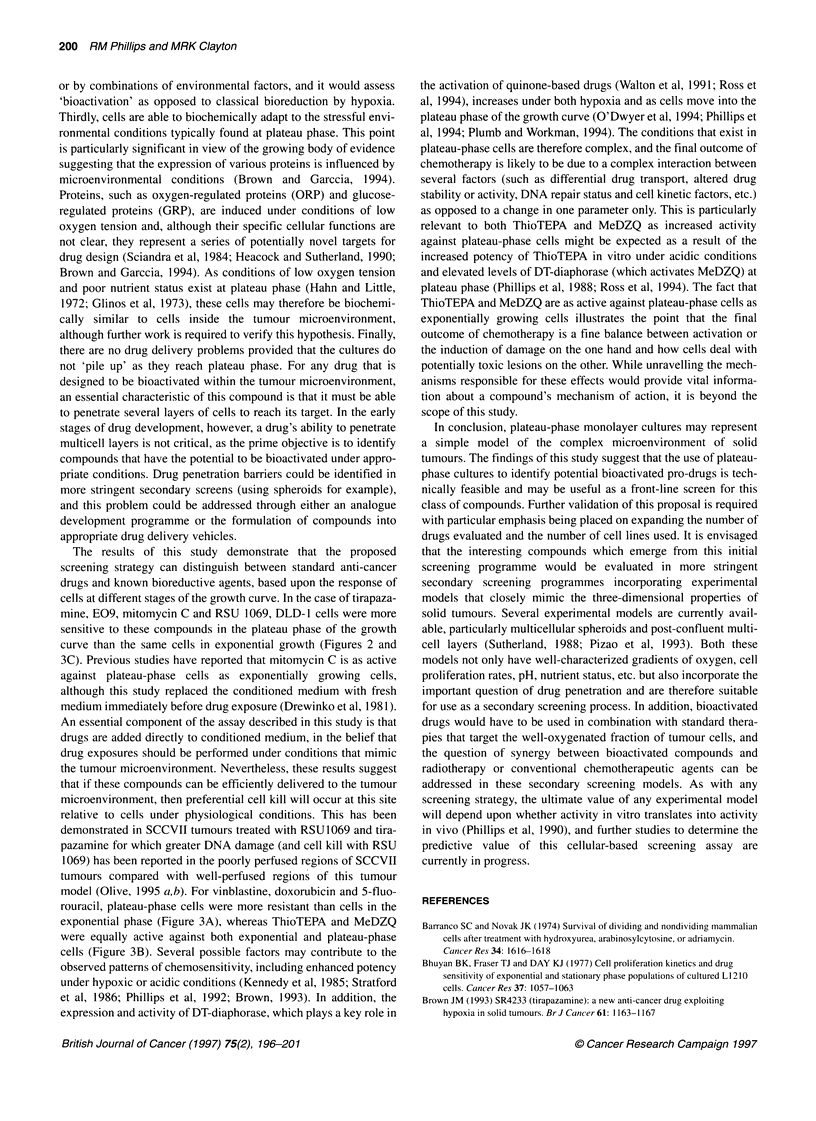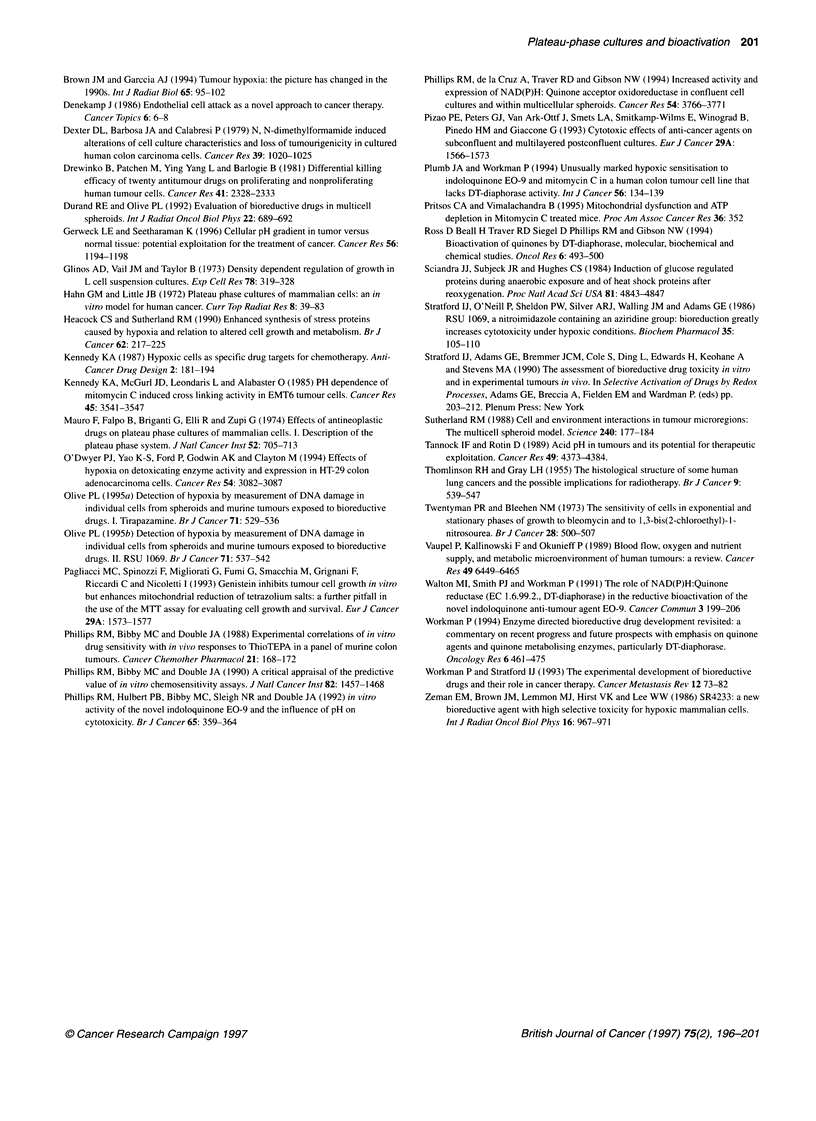# Plateau-phase cultures: an experimental model for identifying drugs which are bioactivated within the microenvironment of solid tumours.

**DOI:** 10.1038/bjc.1997.33

**Published:** 1997

**Authors:** R. M. Phillips, M. R. Clayton

**Affiliations:** Clinical Oncology Unit, University of Bradford, UK.

## Abstract

A commonly used technique for evaluating potential bioreductive drugs is the determination of hypoxic cytotoxicity ratios in vitro. This experimental model, however, does not accurately mimic the tumour microenvironment, as other factors (such as reduced pH, poor nutrient status, low cell proliferation rates and high catabolite concentrations) are not incorporated into the design of the assay. Plateau-phase monolayer cultures possess many of these characteristics, and this study compared the response of plateau-phase and exponentially growing human colon carcinoma cells (DLD-1) with a series of standard and bioreductive compounds. All drugs tested were added directly to conditioned medium and three patterns of chemosensitivity were observed. In the case of doxorubicin, vinblastine and 5-fluorouracil, exponentially growing cells were significantly more responsive than plateau-phase cultures. ThioTEPA and MeDZQ (2,5-diaziridinyl-1, 4-benzoquinone) were equally cytotoxic to both populations of cells. Tirapazamine (SR4233), RSU 1069, mitomycin C and EO-9, however, were preferentially toxic towards plateau-phase compared with exponentially growing cells. While the exact mechanisms responsible for these observations in each case are not known, this study suggests that plateau-phase cultures may prove to be a useful experimental model in the evaluation of drugs designed to work preferentially within the tumour microenvironment.


British Journal of Cancer (1997) 75(2), 196–201
© 1997 Cancer Research Campaign

### Plateau-phase cultures: an experimental model for identifying drugs which are bioactivated within the microenvironment of solid tumours

**RM Phillips and MRK Clayton**

Clinical Oncology Unit, University of Bradford, Bradford BD7 IDP, UK

**Summary** A commonly used technique for evaluating potential bioreductive drugs is the determination of hypoxic cytotoxicity ratios in vitro. This experimental model, however, does not accurately mimic the tumour microenvironment, as other factors (such as reduced pH, poor nutrient status, low cell proliferation rates and high catabolite concentrations) are not incorporated into the design of the assay. Plateau-phase monolayer cultures possess many of these characteristics, and this study compared the response of plateau-phase and exponentially growing human colon carcinoma cells (DLD-1) with a series of standard and bioreductive compounds. All drugs tested were added directly to conditioned medium and three patterns of chemosensitivity were observed. In the case of doxorubicin, vinblastine and 5-fluorouracil, exponentially growing cells were significantly more responsive than plateau-phase cultures. ThioTEPA and MeDZQ (2,5-diaziridinyl-1,4-benzoquinone) were equally cytotoxic to both populations of cells. Tirapazamine (SR4233), RSU 1069, mitomycin C and EO-9, however, were preferentially toxic towards plateau-phase compared with exponentially growing cells. While the exact mechanisms responsible for these observations in each case are not known, this study suggests that plateau-phase cultures may prove to be a useful experimental model in the evaluation of drugs designed to work preferentially within the tumour microenvironment.

**Keywords:** bioreductive drugs; bioactivation; plateau phase; microenvironment

A characteristic feature of many solid tumours is that their vascular supply is inadequate leading to regions of the tumour that are poorly perfused with blood (Thomlinson and Gray, 1955; Denekamp, 1986). This property of solid tumours introduces several factors that reduce the effectiveness of both chemotherapy and radiotherapy. These include gradients of oxygen tension, extracellular pH, nutrients, catabolites, altered expression of biologically and pharmacologically important proteins and cell proliferation rates, all of which vary as a function of distance from a supporting blood vessel (Denekamp, 1986; Sutherland, 1988; Vaupel et al, 1989; Brown and Garccia, 1994). Tumour cells that reside some distance away from a blood vessel therefore exist in a microenvironment which is quite distinct from normal physiological conditions, and there is considerable interest in trying to exploit this physiological difference to obtain a selective therapy for solid tumours (Kennedy, 1987; Tannock and Rotin, 1989; Gerweck and Seetharaman, 1996).

A number of strategies have been employed to try and eradicate these cells, particularly with the development of bioreductive drugs which are prodrugs that are enzymatically reduced to cytotoxic species under hypoxic conditions (Brown and Garccia, 1994; Workman, 1994). Several hypoxic cell cytotoxins have been developed, such as tirapazamine and RSU 1069, which were identified largely on the basis of differential response of cells under air and nitrogen (Zeman et al, 1986; Brown, 1993; Workman and Stratford, 1993). The only variable assayed in these studies is the oxygenation status of the cultures at the time of drug exposure and, in this respect, the model only partly mimics the conditions found within the microenvironment of tumours (Stratford et al, 1990). Drugs which are activated by conditions other than hypoxia (e.g. by acidic pH) may be missed, and there is therefore considerable merit in using experimental models that closely mimic the tumour microenvironment in its entirety to identify compounds of this class. Any compounds identified using such a model could be classified as 'bioactivated drugs' as opposed to bioreductive drugs as several factors may contribute towards cytotoxicity, not just hypoxia. Multicellular spheroids and post-confluent multicell layers (Durand and Olive, 1992; Pizao et al, 1993) closely mimic conditions found within solid tumours, although both techniques are labour intensive and are not suitable as a front line screen for bioactivated pro-drugs. There is therefore a need to develop new experimental models of the tumour microenvironment that retain the simplicity required for the in vitro evaluation of large numbers of potential prodrugs.

In the early 1970s, considerable interest centred upon the use of plateau-phase monolayer cultures as an in vitro model of the non-cycling fraction of cells that exist within solid tumours, and numerous studies have compared the response of plateau-phase cells with cells in exponential growth to both radiotherapy and chemotherapy (Hahn and Little, 1972; Twentyman and Bleehen, 1973; Barranco and Novak, 1974; Drewinko et al, 1981). Plateau-phase cultures have been extensively characterized and, in addition to a reduction in cellular proliferation rates, a decrease in extracellular pH, oxygen tension and nutrient status plus an increase in lactic acid and catabolite concentrations have been described (Hahn and Little, 1972; Glinos et al, 1973; Mauro et al, 1974; Bhuyan et al, 1977). These characteristics of plateau-phase cultures, therefore, closely resemble those found within the

*Received 4 June 1996*
*Revised 8 August 1996*
*Accepted 13 August 1996*

*Correspondence to:* RM Phillips

**Figure 1** Growth characteristics of DLD-1 cells (**A**), the change in extracellular pH (**B**) and mitotic indices (**C**) throughout the growth curve. Each point represents the mean of three independent experiments ± standard deviations hypoxic microenvironment of tumours, whereas cells in exponential growth exist in conditions that are analogous to cells growing close to a blood vessel (i.e. good nutrient status, active cell proliferation, low catabolite concentration, physiological pH, etc.). These differences between plateau-phase and exponentially growing cells provide a foundation for the development of a novel screening strategy, the aim of which is to identify pro-drugs that are bioactivated under conditions which mimic the microenvironment of tumours. Compounds would be selected for further evaluation if they are preferentially toxic towards plateau-phase cells compared with exponentially growing cells. In order to validate this proposed screening strategy, the principal aim of this study is to determine whether or not the sensitivity of DLD-1 human colon carcinoma cells at different stages of the growth curve can distinguish between a series of standard anti-cancer agents and known bioreductive drugs using the selection criteria outlined above.

### MATERIALS AND METHODS

### Culture conditions

DLD-1 human colon carcinoma cells (Dexter et al, 1979) were routinely maintained as monolayer cultures in RPMI-1640 medium (Life Technologies, Paisley, UK) supplemented with 10% fetal calf serum (Advanced Protein Products, Brierly Hill, UK), sodium pyruvate (1 mM, Life Technologies), L-glutamine (1 mM, Life Technologies) and penicillin/streptomycin (100 IU $ml^{-1}$ and 100 $\mu g\ ml^{-1}$, Life Technologies).

### Growth characteristics

DLD-1 cells were seeded at an initial density of $1\times 10^5$ cells per T-25 flask containing 10 ml of growth medium and incubated at 37°C. Total cell number was determined daily by harvesting cells by trypsinization and counting cells using a haemocytometer. Extracellular pH was monitored daily using a pH electrode (Orion Research). Mitotic indices were determined from cells grown on cover slips (placed inside T-25 flasks) which were stained with haematoxylin and eosin. A total of ten fields of view per data point were counted (× 25 objective), and approximately 200–300 cells per data point were counted. All experiments were performed in triplicate.

### Chemosensitivity studies

Drugs used in this study include doxorubicin (Farmitalia Carbo, Barnet, UK), vinblastine (Sigma, Poole, UK), 5-fluorouracil (Sigma), ThioTEPA (a gift from Lederle Laboratories, Gosport, UK), mitomycin C (Kyowa Hakko Kogyo, Tokyo, Japan), EO-9 (The New Drug Development Office of the European Organisation for the Reseach and Treatment of Cancer), tirapazamine (a gift from Sanofi Winthrop, USA), RSU 1069 and MeDZQ (2,5-dimethyl-3,6-diaziridinyl-1,4-benzoquinone; a gift from Dr J Butler, Paterson Institute, Manchester, UK). EO-9 and MeDZQ were dissolved in dimethyl sulphoxide (DMSO), aliquoted and stored at −80°C. All other compounds were dissolved in sterile saline, aliquoted and stored at −80°C. DLD-1 cells were set up at an initial density of $1 \times 10^5$ cells per T-25 flask containing 10 ml of medium and exposed to drugs on days 2 (exponential phase) and days 7 (plateau phase) of the growth curve. All drugs were added directly to the conditioned medium (< 100 µl aliquots) for 1 h (with the exception of vinblastine and tirapazamine, for which drug exposure times of 3 h were used, and 5-fluorouracil, for which 6 h drug exposure times were used). Extended drug exposure times were required to generate full dose–response curves without depleting drug stocks. Following drug exposure, monolayers were washed twice with Hanks' balanced salt solution, and cells were harvested by trypsinization. A total of $1 \times 10^3$ cells in 200 µl of fresh RPMI-1640 medium (not conditioned) were plated into each well of a 96-well plate, and cells were incubated at 37°C for 5 days in an humidified atmosphere containing 5% carbon dioxide. It should be stressed that cells treated on both days 2 and 7 of the growth curve were subjected to identical post-drug exposure recovery periods of 5 days. Following incubation, 20 µl of MTT (5 mg $ml^{-1}$) was added to each well and incubated for a

© Cancer Research Campaign 1997

**Figure 2** The response of DLD-1 cells to doxorubicin (**A**) and EO-9 (**B**) on days 2–8 (inclusive) of the growth curve. Each point represents the mean of three independent experiments ± standard deviations further 4 h. Medium plus MTT was removed from each well, and the formazan crystals were dissolved in 150 µl of DMSO. The absorbance of the resulting solution measured at 550 nm in an ELISA spectrophotometer. Results were expressed in terms of per cent survival, taking the absorbance of control cultures to be 100 % survival. Chemosensitivity was expressed in terms of $IC_{50}$ values (concentration required to kill 50% of the cell population), and all assays were performed in triplicate.

### RESULTS

### Growth characteristics of DLD-1 cells

Growth curves for DLD-1 cells together with the change in extracellular pH and mitotic indices are presented in Figure 1. Following an initial lag phase of 24 h, DLD-1 cells entered exponential growth (population doubling time of 21 h) and reached plateau phase on day 6 (Figure 1A). Extracellular pH decreased from 7.5 ± 0.2 on day 0 to 6.7 ± 0.15 on day 8 of the growth curve (Figure 1B). Mitotic indices reached a peak on day 3 (4.73± 0.38%) and progressively decreased to 0.49 ± 0.21% on day 8 (Figure 1C).

### Chemosensitivity studies

The response of DLD-1 cells exposed to doxorubicin and EO-9 at various stages of the growth curve are presented in Figure 2. As cells progressed through the growth curve, resistance to doxorubicin increased (Figure 2A), particularly between days 4 and 7 of the growth curve. In the case of EO-9, DLD-1 cells progressively became more sensitive as cultures matured into plateau phase (Figure 2B). A comparison between the response of cells in early exponential growth (day 2) and plateau phase (day 7) to a series of compounds is presented in Figure 3. Three patterns of chemosensitivity were observed. In the case of doxorubicin, vinblastine and 5-fluorouracil exponentially growing cells were significantly more responsive than the same cells in plateau phase (Figure 3A). ThioTEPA and MeDZQ were equally effective against plateau-phase cells as exponentially growing cells (Figure 3B). Finally, EO-9, mitomycin C, tirapazamine and RSU 1069 were preferentially cytotoxic towards plateau-phase cultures compared with exponentially growing cells (Figure 3C).

### DISCUSSION

The principal objective of this study was to develop a novel cell-based screening assay that could be used to identify drugs that are activated under conditions which mimic the tumour microenvironment. Plateau-phase cultures closely mimic the microenvironment of tumours, in that cell proliferation rates are reduced, the extracellular pH is acidic, nutrient levels are low, catabolite concentrations are high and oxygen tension is low (Figure 1; Hahn and Little, 1972; Glinos et al, 1973). The assay described in this study is therefore quite distinct from currently available assays used to evaluate bioreductive drugs, in that it assesses a compound's ability to be activated under conditions that mimic several properties of the tumour microenvironment, not just hypoxia. The ability to kill non-proliferating cells is essential if this class of compounds is going to be effective in the treatment of solid tumours and this is a key feature of the proposed screening programme.

The use of plateau-phase cultures has several additional advantages over other models of the tumour microenvironment, such as multicellular spheroids. Firstly, it is a technically simple model in which drugs are added directly to the conditioned medium and chemosensitivity assessed using conventional assays, such as clonogenic or MTT assays. In the case of MTT assays, however, problems can occur with drugs that are either mitochondrial poisons or cause an increase in mitochondrial density or activity (Pagliacci et al, 1993; Pritsos and Vimalachandra, 1995). Provided that short drug exposures are followed by a long drug-free recovery period (5 days in this study), then many of these problems can be eliminated. Secondly, it would detect drugs that are activated by conditions other than hypoxia alone, such as low pH,

© Cancer Research Campaign 1997

**Figure 3** Comparison between the response of exponentially growing (E, open bars) and plateau-phase (P, hatched bars) DLD-1 cells to doxorubicin, vinblastine, 5-fluorouracil, MeDZQ, ThioTEPA, RSU 1069, tirapazamine, mitomycin C and EO-9. Each point represents the mean of three independent experiments ± standard deviations

*© Cancer Research Campaign 1997*

or by combinations of environmental factors, and it would assess 'bioactivation' as opposed to classical bioreduction by hypoxia. Thirdly, cells are able to biochemically adapt to the stressful environmental conditions typically found at plateau phase. This point is particularly significant in view of the growing body of evidence suggesting that the expression of various proteins is influenced by microenvironmental conditions (Brown and Garccia, 1994). Proteins, such as oxygen-regulated proteins (ORP) and glucose-regulated proteins (GRP), are induced under conditions of low oxygen tension and, although their specific cellular functions are not clear, they represent a series of potentially novel targets for drug design (Sciandra et al, 1984; Heacock and Sutherland, 1990; Brown and Garccia, 1994). As conditions of low oxygen tension and poor nutrient status exist at plateau phase (Hahn and Little, 1972; Glinos et al, 1973), these cells may therefore be biochemically similar to cells inside the tumour microenvironment, although further work is required to verify this hypothesis. Finally, there are no drug delivery problems provided that the cultures do not 'pile up' as they reach plateau phase. For any drug that is designed to be bioactivated within the tumour microenvironment, an essential characteristic of this compound is that it must be able to penetrate several layers of cells to reach its target. In the early stages of drug development, however, a drug's ability to penetrate multicell layers is not critical, as the prime objective is to identify compounds that have the potential to be bioactivated under appropriate conditions. Drug penetration barriers could be identified in more stringent secondary screens (using spheroids for example), and this problem could be addressed through either an analogue development programme or the formulation of compounds into appropriate drug delivery vehicles.

The results of this study demonstrate that the proposed screening strategy can distinguish between standard anti-cancer drugs and known bioreductive agents, based upon the response of cells at different stages of the growth curve. In the case of tirapazamine, EO9, mitomycin C and RSU 1069, DLD-1 cells were more sensitive to these compounds in the plateau phase of the growth curve than the same cells in exponential growth (Figures 2 and 3C). Previous studies have reported that mitomycin C is as active against plateau-phase cells as exponentially growing cells, although this study replaced the conditioned medium with fresh medium immediately before drug exposure (Drewinko et al, 1981). An essential component of the assay described in this study is that drugs are added directly to conditioned medium, in the belief that drug exposures should be performed under conditions that mimic the tumour microenvironment. Nevertheless, these results suggest that if these compounds can be efficiently delivered to the tumour microenvironment, then preferential cell kill will occur at this site relative to cells under physiological conditions. This has been demonstrated in SCCVII tumours treated with RSU1069 and tirapazamine for which greater DNA damage (and cell kill with RSU 1069) has been reported in the poorly perfused regions of SCCVII tumours compared with well-perfused regions of this tumour model (Olive, 1995 *a,b*). For vinblastine, doxorubicin and 5-fluorouracil, plateau-phase cells were more resistant than cells in the exponential phase (Figure 3A), whereas ThioTEPA and MeDZQ were equally active against both exponential and plateau-phase cells (Figure 3B). Several possible factors may contribute to the observed patterns of chemosensitivity, including enhanced potency under hypoxic or acidic conditions (Kennedy et al, 1985; Stratford et al, 1986; Phillips et al, 1992; Brown, 1993). In addition, the expression and activity of DT-diaphorase, which plays a key role in the activation of quinone-based drugs (Walton et al, 1991; Ross et al, 1994), increases under both hypoxia and as cells move into the plateau phase of the growth curve (O'Dwyer et al, 1994; Phillips et al, 1994; Plumb and Workman, 1994). The conditions that exist in plateau-phase cells are therefore complex, and the final outcome of chemotherapy is likely to be due to a complex interaction between several factors (such as differential drug transport, altered drug stability or activity, DNA repair status and cell kinetic factors, etc.) as opposed to a change in one parameter only. This is particularly relevant to both ThioTEPA and MeDZQ as increased activity against plateau-phase cells might be expected as a result of the increased potency of ThioTEPA in vitro under acidic conditions and elevated levels of DT-diaphorase (which activates MeDZQ) at plateau phase (Phillips et al, 1988; Ross et al, 1994). The fact that ThioTEPA and MeDZQ are as active against plateau-phase cells as exponentially growing cells illustrates the point that the final outcome of chemotherapy is a fine balance between activation or the induction of damage on the one hand and how cells deal with potentially toxic lesions on the other. While unravelling the mechanisms responsible for these effects would provide vital information about a compound's mechanism of action, it is beyond the scope of this study.

In conclusion, plateau-phase monolayer cultures may represent a simple model of the complex microenvironment of solid tumours. The findings of this study suggest that the use of plateau-phase cultures to identify potential bioactivated pro-drugs is technically feasible and may be useful as a front-line screen for this class of compounds. Further validation of this proposal is required with particular emphasis being placed on expanding the number of drugs evaluated and the number of cell lines used. It is envisaged that the interesting compounds which emerge from this initial screening programme would be evaluated in more stringent secondary screening programmes incorporating experimental models that closely mimic the three-dimensional properties of solid tumours. Several experimental models are currently available, particularly multicellular spheroids and post-confluent multicell layers (Sutherland, 1988; Pizao et al, 1993). Both these models not only have well-characterized gradients of oxygen, cell proliferation rates, pH, nutrient status, etc. but also incorporate the important question of drug penetration and are therefore suitable for use as a secondary screening process. In addition, bioactivated drugs would have to be used in combination with standard therapies that target the well-oxygenated fraction of tumour cells, and the question of synergy between bioactivated compounds and radiotherapy or conventional chemotherapeutic agents can be addressed in these secondary screening models. As with any screening strategy, the ultimate value of any experimental model will depend upon whether activity in vitro translates into activity in vivo (Phillips et al, 1990), and further studies to determine the predictive value of this cellular-based screening assay are currently in progress.

### REFERENCES

Barranco SC and Novak JK (1974) Survival of dividing and nondividing mammalian cells after treatment with hydroxyurea, arabinosylcytosine, or adriamycin. *Cancer Res* **34**: 1616–1618

Bhuyan BK, Fraser TJ and DAY KJ (1977) Cell proliferation kinetics and drug sensitivity of exponential and stationary phase populations of cultured L1210 cells. *Cancer Res* **37**: 1057–1063

Brown JM (1993) SR4233 (tirapazamine): a new anti-cancer drug exploiting hypoxia in solid tumours. *Br J Cancer* **61**: 1163–1167

 © Cancer Research Campaign 1997

Brown JM and Garccia AJ (1994) Tumour hypoxia: the picture has changed in the 1990s. *Int J Radiat Biol* **65**: 95–102

Denekamp J (1986) Endothelial cell attack as a novel approach to cancer therapy. *Cancer Topics* **6**: 6–8

Dexter DL, Barbosa JA and Calabresi P (1979) N, N-dimethylformamide induced alterations of cell culture characteristics and loss of tumourigenicity in cultured human colon carcinoma cells. *Cancer Res* **39**: 1020–1025

Drewinko B, Patchen M, Ying Yang L and Barlogie B (1981) Differential killing efficacy of twenty antitumour drugs on proliferating and nonproliferating human tumour cells. *Cancer Res* **41**: 2328–2333

Durand RE and Olive PL (1992) Evaluation of bioreductive drugs in multicell spheroids. *Int J Radiat Oncol Biol Phys* **22**: 689–692

Gerweck LE and Seetharaman K (1996) Cellular pH gradient in tumor versus normal tissue: potential exploitation for the treatment of cancer. *Cancer Res* **56**: 1194–1198

Glinos AD, Vail JM and Taylor B (1973) Density dependent regulation of growth in L cell suspension cultures. *Exp Cell Res* **78**: 319–328

Hahn GM and Little JB (1972) Plateau phase cultures of mammalian cells: an *in vitro* model for human cancer. *Curr Top Radiat Res* **8**: 39–83

Heacock CS and Sutherland RM (1990) Enhanced synthesis of stress proteins caused by hypoxia and relation to altered cell growth and metabolism. *Br J Cancer* **62**: 217–225

Kennedy KA (1987) Hypoxic cells as specific drug targets for chemotherapy. *Anti-Cancer Drug Design* **2**: 181–194

Kennedy KA, McGurl JD, Leondaris L and Alabaster O (1985) PH dependence of mitomycin C induced cross linking activity in EMT6 tumour cells. *Cancer Res* **45**: 3541–3547

Mauro F, Falpo B, Briganti G, Elli R and Zupi G (1974) Effects of antineoplastic drugs on plateau phase cultures of mammalian cells. I. Description of the plateau phase system. *J Natl Cancer Inst* **52**: 705–713

O'Dwyer PJ, Yao K-S, Ford P, Godwin AK and Clayton M (1994) Effects of hypoxia on detoxicating enzyme activity and expression in HT-29 colon adenocarcinoma cells. *Cancer Res* **54**: 3082–3087

Olive PL (1995*a*) Detection of hypoxia by measurement of DNA damage in individual cells from spheroids and murine tumours exposed to bioreductive drugs. I. Tirapazamine. *Br J Cancer* **71**: 529–536

Olive PL (1995*b*) Detection of hypoxia by measurement of DNA damage in individual cells from spheroids and murine tumours exposed to bioreductive drugs. II. RSU 1069. *Br J Cancer* **71**: 537–542

Pagliacci MC, Spinozzi F, Migliorati G, Fumi G, Smacchia M, Grignani F, Riccardi C and Nicoletti I (1993) Genistein inhibits tumour cell growth *in vitro* but enhances mitochondrial reduction of tetrazolium salts: a further pitfall in the use of the MTT assay for evaluating cell growth and survival. *Eur J Cancer* **29A**: 1573–1577

Phillips RM, Bibby MC and Double JA (1988) Experimental correlations of *in vitro* drug sensitivity with *in vivo* responses to ThioTEPA in a panel of murine colon tumours. *Cancer Chemother Pharmacol* **21**: 168–172

Phillips RM, Bibby MC and Double JA (1990) A critical appraisal of the predictive value of *in vitro* chemosensitivity assays. *J Natl Cancer Inst* **82**: 1457–1468

Phillips RM, Hulbert PB, Bibby MC, Sleigh NR and Double JA (1992) *in vitro* activity of the novel indoloquinone EO-9 and the influence of pH on cytotoxicity. *Br J Cancer* **65**: 359–364

Phillips RM, de la Cruz A, Traver RD and Gibson NW (1994) Increased activity and expression of NAD(P)H: Quinone acceptor oxidoreductase in confluent cell cultures and within multicellular spheroids. *Cancer Res* **54**: 3766–3771

Pizao PE, Peters GJ, Van Ark-Ottf J, Smets LA, Smitkamp-Wilms E, Winograd B, Pinedo HM and Giaccone G (1993) Cytotoxic effects of anti-cancer agents on subconfluent and multilayered postconfluent cultures. *Eur J Cancer* **29A**: 1566–1573

Plumb JA and Workman P (1994) Unusually marked hypoxic sensitisation to indoloquinone EO-9 and mitomycin C in a human colon tumour cell line that lacks DT-diaphorase activity. *Int J Cancer* **56**: 134–139

Pritsos CA and Vimalachandra B (1995) Mitochondrial dysfunction and ATP depletion in Mitomycin C treated mice. *Proc Am Assoc Cancer Res* **36**: 352

Ross D Beall H Traver RD Siegel D Phillips RM and Gibson NW (1994) Bioactivation of quinones by DT-diaphorase, molecular, biochemical and chemical studies. *Oncol Res* **6**: 493–500

Sciandra JJ, Subjeck JR and Hughes CS (1984) Induction of glucose regulated proteins during anaerobic exposure and of heat shock proteins after reoxygenation. *Proc Natl Acad Sci USA* **81**: 4843–4847

Stratford IJ, O'Neill P, Sheldon PW, Silver ARJ, Walling JM and Adams GE (1986) RSU 1069, a nitroimidazole containing an aziridine group: bioreduction greatly increases cytotoxicity under hypoxic conditions. *Biochem Pharmacol* **35**: 105–110

Stratford IJ, Adams GE, Bremmer JCM, Cole S, Ding L, Edwards H, Keohane A and Stevens MA (1990) The assessment of bioreductive drug toxicity *in vitro* and in experimental tumours *in vivo*. In *Selective Activation of Drugs by Redox Processes*, Adams GE, Breccia A, Fielden EM and Wardman P. (eds) pp. 203–212. Plenum Press: New York

Sutherland RM (1988) Cell and environment interactions in tumour microregions: The multicell spheroid model. *Science* **240**: 177–184

Tannock IF and Rotin D (1989) Acid pH in tumours and its potential for therapeutic exploitation. *Cancer Res* **49**: 4373–4384.

Thomlinson RH and Gray LH (1955) The histological structure of some human lung cancers and the possible implications for radiotherapy. *Br J Cancer* **9**: 539–547

Twentyman PR and Bleehen NM (1973) The sensitivity of cells in exponential and stationary phases of growth to bleomycin and to 1,3-bis(2-chloroethyl)-1-nitrosourea. *Br J Cancer* **28**: 500–507

Vaupel P, Kallinowski F and Okunieff P (1989) Blood flow, oxygen and nutrient supply, and metabolic microenvironment of human tumours: a review. *Cancer Res* **49** 6449–6465

Walton MI, Smith PJ and Workman P (1991) The role of NAD(P)H:Quinone reductase (EC 1.6.99.2., DT-diaphorase) in the reductive bioactivation of the novel indoloquinone anti-tumour agent EO-9. *Cancer Commun* **3** 199–206

Workman P (1994) Enzyme directed bioreductive drug development revisited: a commentary on recent progress and future prospects with emphasis on quinone agents and quinone metabolising enzymes, particularly DT-diaphorase. *Oncology Res* **6** 461–475

Workman P and Stratford IJ (1993) The experimental development of bioreductive drugs and their role in cancer therapy. *Cancer Metastasis Rev* **12** 73–82

Zeman EM, Brown JM, Lemmon MJ, Hirst VK and Lee WW (1986) SR4233: a new bioreductive agent with high selective toxicity for hypoxic mammalian cells. *Int J Radiat Oncol Biol Phys* **16**: 967–971

© Cancer Research Campaign 1997